# Effects of Isosakuranetin on Pharmacokinetic Changes of Tofacitinib in Rats with *N*-Dimethylnitrosamine-Induced Liver Cirrhosis

**DOI:** 10.3390/pharmaceutics14122684

**Published:** 2022-12-01

**Authors:** Sung Hun Bae, Hyeon Gyeom Choi, So Yeon Park, Sun-Young Chang, Hyoungsu Kim, So Hee Kim

**Affiliations:** 1 College of Pharmacy and Research Institute of Pharmaceutical Science and Technology, Ajou University, 206 Worldcup-ro, Yeongtong-gu, Suwon 16499, Republic of Korea; 2 Department of Biohealth Regulatory Science, Graduate School of Ajou University, 206 Worldcup-ro, Yeongtong-gu, Suwon 16499, Republic of Korea

**Keywords:** tofacitinib, liver cirrhosis, isosakuranetin, pharmacokinetics, CYP3A1/2, CYP2C11, PXR, CAR

## Abstract

Tofacitinib, a Janus kinase 1 and 3 inhibitor, is used to treat rheumatoid arthritis. It is mainly metabolized by the cytochromes p450 (CYP) 3A1/2 and CYP2C11 in the liver. Chronic inflammation eventually leads to cirrhosis in patients with rheumatoid arthritis. Isosakuranetin (ISN), a component of *Citrus aurantium* L., has hepatoprotective effects in rats. This study was performed to determine the effects of ISN on the pharmacokinetics of tofacitinib in rats with *N*-dimethylnitrosamine-induced liver cirrhosis (LC). After intravenous administration of 10 mg/kg tofacitinib to control (CON), LC, and LC treated with ISN (LC-ISN) rats, the total area under the plasma concentration–time curves (AUC) from time zero to infinity increased by 158% in LC rats compared to those in CON rats; however, the AUC of LC-ISN rats decreased by 35.1% compared to that of LC rat. Similar patterns of AUC changes were observed in the LC and LC-ISN rats after oral administration of 20 mg/kg tofacitinib. These results can be attributed to decreased non-renal clearance (CL_NR_) and intestinal intrinsic clearance (CL_int_) in the LC rats and increased intestinal and hepatic CL_int_ in the LC-ISN rats. Our findings imply that ISN treatment in LC rats restored the decrease in either CL_NR_ or CL_int_, or both, through increased hepatic and intestinal expression of CYP3A1/2 and CYP2C11, which is regulated by the induction of pregnane X receptor (PXR) and constitutive androstane receptor (CAR).

## 1. Introduction

**Figure 1:**
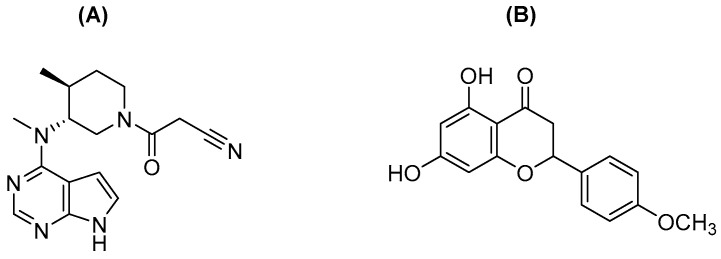
Chemical structure of tofacitinib (**A**) and isosakuranetin (**B**).

Tofacitinib (Figure 1A), 3-(4-methyl-3-(methyl(7H-pyrrolo[2,3-d]pyrimidin-4yl)amino)piperidin-1-yl)-3-oxopropanenitrile, is a Janus kinase (JAK) 1 and 3 inhibitor developed to treat moderate to severe rheumatoid arthritis that does not respond to methotrexate therapy [1]. It suppresses the JAK-signal transducer and activator of transcription signaling pathways in patients with rheumatoid arthritis [2]. Tofacitinib modulates several immune responses and blocks inflammatory mediators, such as interleukins (IL) −2, −4, −7, −9, −15, and −21 [1,3,4]. Recently, tofacitinib has been approved as an oral drug for inflammatory bowel diseases, such as ulcerative colitis [5]. In addition, tofacitinib has been clinically evaluated for use in psoriasis [6,7] alopecia [8] atopic dermatitis [9], and ankylosing spondylitis [10].

In human studies, approximately 70% of the oral tofacitinib dose is metabolized by *N*-demethylation and oxidation via the hepatic microsomal cytochromes p450 (CYP) 3A4 and CYP2C19, and 30% of the remaining tofacitinib is excreted by the kidneys in its unmetabolized form [11,12]. In rats, the hepatic intestinal first-pass effects were 42.0% and 46.1%, respectively, after intraportal and intraduodenal administration of tofacitinib [13].

The pharmacokinetics of drugs can be affected by liver impairment owing to the considerable hepatic and intestinal first-pass metabolism. In patients with rheumatoid arthritis, chronic inflammation may spread to the liver, eventually leading to cirrhosis over time [14]. Damaged hepatocytes have a strong regenerative ability to a certain extent. However, prolonged hepatocellular damage may lead to hepatitis and later develop into irreversible liver cirrhosis (LC) [15]. LC, in its terminal stage of liver disease, can affect drug metabolism [16].

Isosakuranetin (ISN), an *O*-methylated flavonoid (Figure 1B), is an aglycone of poncirin found in sweet oranges [17]. It inhibits the transient receptor potential melastatin 3 (TRPM3), resulting in thermal nociception [18]. Its biological activities include antioxidant, anti-inflammatory, and antinociceptive effects [19]. Nonetheless, limited information is available on the effects of ISN on LC and the pharmacokinetics of drugs.

The purpose of this study was to evaluate whether ISN could prevent or attenuate LC and investigate the pharmacokinetic changes in LC rats induced by *N*-dimethylnitrosamine (DMN) and LC rats with treatment of ISN (LC-ISN) following intravenous and oral administration of tofacitinib. The microsomal activities of tofacitinib and CYP enzymes in the liver and intestine were also evaluated.

## 2. Materials and Methods

### 2.1. Chemicals

Tofacitinib citrate (MW:504.49) and hydrocortisone (MW:362.46) as an internal standard for high-performance liquid chromatography (HPLC) were obtained from Sigma Aldrich (St. Louis, MO, USA). ISN was donated by Professor Hyungsu Kim (Ajou University, Suwon, Republic of Korea). Ethyl acetate and acetonitrile for the quantification of tofacitinib by HPLC analysis were purchased from J.T. Baker (Phillipsburg, NJ, USA). DMN was supplied by Tokyo Chemical Industry (Tokyo, Japan), and β-cyclodextrin was obtained from Wako (Osaka, Japan). The NaCl-injectable solution and heparin were purchased from JW Pharmaceutical Corporation (Seoul, Republic of Korea). Antibodies against CYP3A1/2, CYP2E1, CYP2C11, CYP2D1, CYP2B1/2, and CYP1A1/2 were donated by Detroit R&D Inc. (Detroit, MI, USA), and primary antibodies against P-gp, pregnane X receptor (PXR), and constitutive androstane receptor (CAR) were purchased from Abcam (Cambridge, UK). β-Actin, used as a loading control, was supplied by Cell Signaling Technology (Beverly, MA, USA). Goat, rabbit, and mouse secondary antibodies were purchased from Bio-Rad (Hercules, CA, USA). All the reagents and other chemicals were of HPLC or analytical grade and were used without further purification.

### 2.2. Animals

Male Sprague–Dawley rats (3 weeks old and 60–70 g weight) were obtained from OrientBio (Seongnam, Republic of Korea). They were maintained at a relative humidity of 50 ± 5% through air purification and a temperature of 22 ± 1 °C in 12 h day (7:00–19:00 h) and night (19:00–7:00 h) cycles at the Laboratory Animal Research Center of Ajou University Medical Center (Suwon, Korea). Rats were allowed free access to water and food ad libitum during the experimental period of 6 weeks. The rat experiments and protocols were reviewed and approved by the Institutional Animal Care and Use Committee of the Laboratory Animal Research Center of Ajou University Medical Center (IACUC No. 2020-0013, 26 August 2020).

### 2.3. Induction of Liver Cirrhosis

DMN induces LC in rats through the destruction of hepatic parenchymal cell, connective tissue formation, and nodule regeneration [20], which eventually increases mortality. The characteristics of LC induced by DMN are similar to the clinical characteristics exhibited by patients with LC [20]. The DMN-induced LC rat model showed pathological alterations, including extensive micronodular cirrhosis with regenerative hepatocellular changes [21] and high mortality during the experiment [22].

Before the start of the 6-week treatment, the rats were randomly assigned to one of three groups (hereafter referred to as CON, LC, and LC-ISN). To induce LC, DMN was dissolved in 0.9% NaCl-injectable solution and administered intraperitoneally at a dose of 10 mg/kg to the LC rats on three consecutive days per week for six weeks [23]. ISN was suspended in 0.5% carboxymethylcellulose (CMC) sodium and administered orally at a dose of 15 mg/kg [24] daily to the LC-ISN rats for six weeks. CON rats were administered the same volumes of 0.9% NaCl-injectable solution and 0.5% CMC as for the LC and LC-ISN rats, respectively, during the 6-week experimental period.

### 2.4. Preliminary Study

A preliminary study was conducted on the CON, LC, and LC-ISN rats (*n* = 3, each group) to measure the liver and kidney function. Plasma was collected from each group to estimate albumin, alkaline phosphatase (ALP), glutamate pyruvate transaminase (GPT), glutamate oxaloacetate transaminase (GOT), serum creatinine, urea nitrogen, cholesterol, and total protein levels (Green Cross Reference Lab, Seoul, Republic of Korea). Assuming that the function of the kidney was stable during the experimental period, 24 h urine samples were collected to measure the urine volumes and creatinine levels to estimate creatinine clearance (CL_CR_). The CL_CR_ value was obtained by dividing the total amount of creatinine excreted in urine over 24 h by the area under the plasma concentration–time curve (AUC) of creatinine from 0 to 24 h (AUC_0–24 h_). A small portion of the liver and kidneys from the rats in each group were soaked in 10% neutral buffered formalin (BBC Biochemical, Mount Vernon, WA, USA) for histological analysis with H&E staining.

### 2.5. Rat Plasma Protein Binding of Tofacitinib

Fresh plasma obtained from the CON, LC, and LC-ISN rats (*n* = 3 per group) was used for the plasma protein binding studies of tofacitinib using equilibrium dialysis [25]. Rat plasma (1 mL) from each group was dialyzed against 1 mL of isotonic Sørensen phosphate buffer (pH 7.4) containing 3% dextran (“the buffer”) via a Spectra/Por 2 membrane (molecular weight cutoff: 12,000–14,000 Da; Spectrum Medical Industries, Los Angeles, CA, USA). To shorten the time to reach equilibrium, 10 μg/mL tofacitinib was added into the plasma section [26,27]. After incubation in a water bath at 37 °C at a rate of 50 oscillations per min for 24 h, 50 μL aliquots were withdrawn from the rat plasma and the buffer sections, and the concentrations of tofacitinib in the rat plasma (*C_P_*) and buffer (*C_B_*) were measured using HPLC [28]. The binding percentage (%) was calculated as follows:

(1) $$\mathrm{Binding}\;\%=\frac{C_{P}-C_{B}}{C_{P}}\times 100$$

### 2.6. Intravenous and Oral Administration of Tofacitinib

All surgical procedures were performed following anesthesia with 100 mg/kg ketamine according to a previously reported method [13,29]. For the intravenous study, the carotid artery and jugular vein were cannulated for blood sampling and drug administration, respectively. For the oral study, rats were restrained from food overnight but were allowed to drink water ad libitum. Only the carotid artery was cannulated for the blood sampling. After sufficient recovery from anesthesia for 3–4 h, the experiments were commenced [29].

For intravenous administration, tofacitinib (dissolved in 0.9% NaCl-injectable solution containing 0.5% β-cyclodextrin) was infused at a dose of 10 mg/kg via the jugular veins for 1 min to the CON (*n* = 5), LC (*n* = 6), and LC-ISN (*n* = 7) rats. Blood samples (150 µL) were collected via the carotid artery at 0 (before the infusion of tofacitinib), 1, 5, 15, 30, 45, 60, 90, 120, 180, 240, 360, 480, 600, and 720 min after infusion. For oral administration, tofacitinib was administered to the CON (*n* = 6), LC (*n* = 7), and LC-ISN (*n* = 7) rats at a dose of 20 mg/kg. Blood samples (150 μL) were collected via the carotid artery at 0 (prior to the administration of tofacitinib), 5, 15, 30, 45, 60, 90, 120, 180, 240, 360, 480, 600, and 720 min after administration. A heparinized 0.9% NaCl-injectable solution (10 IU/mL) was immediately flushed into the carotid artery after collecting the blood samples to prevent blood clotting. Each sample was immediately centrifuged at 8000× *g* for 1 min, and 50 μL plasma was collected and stored at −80 °C until HPLC analysis of tofacitinib [28].

Urine samples were also collected for 24 h after the administration of tofacitinib, and each metabolic cage was rinsed with 20 mL of distilled water, and the rinsings were combined with the 24 h urine samples. The volume of the combined urine was then measured, and two 100 μL aliquot samples were collected. At the same time, the rat abdomen was surgically opened, and the whole gastrointestinal tract was collected in a beaker containing 50 mL methanol and cut into small pieces. After even mixing, 50 µL of the supernatant was collected. All aliquots of urine and gastrointestinal tract samples were stored at −80 °C in a deep freezer for subsequent HPLC analysis of tofacitinib [28].

### 2.7. Tissue Distribution of Tofacitinib

Tofacitinib (10 mg/kg) was intravenously administered to CON, LC, and LC-ISN rats (*n* = 3, each group). After 30 min, the maximum amount of blood was obtained from each rat through the carotid artery, and the blood samples were immediately centrifuged to collect plasma. Approximately 1 *g* of each tissue, including fat, brain, kidney, heart, liver, large intestine, mesentery, lung, small intestine, muscle, stomach, and spleen was collected and washed with phosphate-buffered solution (pH 7.4) to remove residual blood. The tissue sample was homogenized (T25 Ultra-Turrax, IKA Labortechnik, Staufen, Germany) using four volumes of homogenizing buffer and immediately centrifuged for 3 min at 8000*× g*. An aliquot of supernatant (50 μL) was collected and stored at −80 °C in a deep freezer for subsequent HPLC analysis of tofacitinib [28].

### 2.8. Measurement of V_max_, K_m_ and CL_int_

Enzyme kinetics in the hepatic and intestinal microsomes were determined according to a previously reported method [30,31]. Microsomal protein concentrations in the liver and intestine were measured using bicinchoninic acid assay. Microsomes (1 mg/mL protein), 1 μL dimethyl sulfoxide containing tofacitinib (5, 10, 20, 50, 100, 200, and 500 μM), and a nicotinamide adenine dinucleotide phosphate hydrogen-generating system (Corning Inc., Corning, NY, USA) were used in the in vitro metabolic system. The total volume was adjusted to 1 mL by adding 0.1 M phosphate-buffered solution (pH 7.4) to the system, and the samples were incubated for 15 min in a water-bath shaker at 37 °C at the rate of 50 oscillations per min. The reaction was completed by adding 2 volumes of methanol. Subsequently, two 50 μL aliquots of each reaction mixture were collected. Kinetic constants, maximum velocity (*V*_max_), and apparent Michaelis–Menten constant (*K*_m_; the concentration of tofacitinib at one-half of *V*_max_) were measured using a Lineweaver–Burk plot followed by nonlinear regression analysis [30,32] using Prism 5 (GraphPad Software Inc., San Diego, CA, USA). The intrinsic clearance (CL_int_) for tofacitinib disappearance in hepatic and intestinal microsomes was calculated by dividing the *V*_max_ by *K*_m_ [30,32]

### 2.9. Immunoblot Analysis

For immunoblot analysis, hepatic and intestinal microsomal proteins (20–40 μg protein per lane) were resolved by 10% sodium dodecyl sulfate polyacrylamide gel electrophoresis (SDS-PAGE). Proteins were transferred onto nitrocellulose membranes and blocked with 5% skim milk in tris-buffered saline (TBS) containing 0.1% Tween 20 (TBS-T) for 1 h [33]. For immunodetection, membranes were incubated with primary antibodies against P-gp, CAR, PXR, β-actin, CYP1A1/2, CYP2B1/2, CYP2C11, CYP2D1, CYP2E1, and CYP3A1/2 that were diluted in TBS-T containing 5% bovine serum albumin at 1:2000 with overnight gentle shaking at 4 °C. The membranes were then incubated with horseradish peroxidase-conjugated rabbit, mouse, or goat secondary antibody diluted at 1:10,000 with 5% skim milk in TBS-T at room temperature for 1 h. Protein bands were detected by enhanced chemiluminescence (Bio-Rad) using an Image Quant LAS 4000 Mini (GE Healthcare Life Sciences, Piscataway, NJ, USA). Band densities were determined using ImageJ 1.45s software (NIH, Bethesda, MA, USA) with β-actin as a loading control [31].

### 2.10. HPLC Analysis

Biological sample preparation for the HPLC analysis of tofacitinib was conducted according to a previously reported method [28]. Briefly, an aliquot of the biological sample (50 μL) was mixed with hydrocortisone (1 μL, 5 mg/mL) as an internal standard and 20% ammonia solution (20 μL) for 30 s. The mixture was then extracted with ethyl acetate (750 μL), and the organic layer was collected and dried under a mild flow of nitrogen gas at 40 °C (Eyela, Tokyo, Japan). The residue was then reconstituted with 20% acetonitrile (130 μL), and the supernatant (50 μL) was analyzed using an HPLC system.

The concentration of tofacitinib in the biological samples was determined using a Prominence LC-20A HPLC system (Shimadzu, Kyoto, Japan) equipped with a reversed-phase column (C_18_; 250 mm × 4.6 mm, 5 μm; Young Jin Biochrom, Seongnam, Republic of Korea) and a UV detector set at 287 nm. The mobile phase consisted of 10 mM ammonium acetate buffer (pH 5.0) and acetonitrile in a 69.5:30.5 (*v*/*v*) ratio, with a flow rate of 1 mL/min. The retention times of tofacitinib and hydrocortisone were approximately 7.21 and 11.3 min, respectively. The lower limits of quantitation of tofacitinib in rat plasma and urine were 0.01 and 0.1 μg/mL, respectively. The intra-day assay precisions (coefficients of variation) ranged from 3.69–5.88% in rat plasma and 4.21–6.18% in urine, and the corresponding inter-day assay precisions were 5.06% and 5.46% in rat plasma and urine, respectively [28].

### 2.11. Pharmacokinetic Analysis

Pharmacokinetic parameters were estimated using the standard method [34] by non-compartmental analysis (WinNonlin; Pharsight Corporation, Mountain View, CA, USA). AUC values were calculated using the trapezoidal rule–extrapolation method [35]. The peak plasma concentration (*C*_max_) and time to reach *C*_max_ (*T*_max_) were measured directly from the plasma concentration–time curves. The extent of absolute oral bioavailability (*F*) value of tofacitinib was calculated by dividing the dose-normalized AUC_oral_ by the AUC_IV_, where AUC_oral_ and AUC_IV_ were the AUCs after oral and intravenous administration, respectively. The glomerular filtration rate was measured by calculating the CL_CR_, assuming that the kidney function was stable during the experimental period.

### 2.12. Statistical Analysis

A *p*-value < 0.05 was considered to be significantly different after analysis of variance followed by Tukey’s post-test for comparison among the three means. All data were expressed as the mean ± standard deviation.

## 3. Results

### 3.1. Preliminary Study

**Figure 2:**
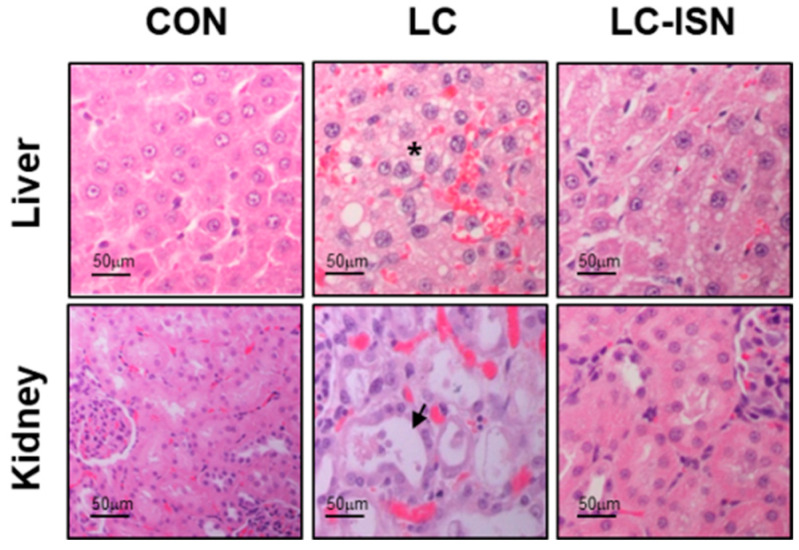
Liver and kidney biopsies in CON, LC and LC-ISN rats. Star symbol indicates tissue damages including necrosis and fibrosis in the liver. Arrow symbol indicates cell death and tissue damage in the kidney. CON, control; LC, liver cirrhosis induced by *N*-dimethylnitrosamine; LC-ISN, LC with treatment of isosakuranetin.

Body weight, biochemical data, plasma protein binding of tofacitinib, CL_CR_, and relative liver and kidney weights of CON, LC, and LC ISN rats are presented in Table 1. LC rats had impaired liver function, and GOT and GPT levels significantly increased by 182% and 137%, respectively, compared to those in CON rats. Total cholesterol and ALP levels were also significantly increased by 83.9% and 455%, respectively, in LC rats compared to those in CON rats. The LC rats had lower total protein (40.3%) and albumin (44.1%) levels than CON rats. Urea nitrogen was comparable, but CL_CR_ decreased by 53.2% in LC rats compared to that in CON rats. The protein binding of tofacitinib in rat plasma decreased in LC and LC-ISN rats by 28.3% and 21.5%, respectively. All biochemical parameters, except for albumin, were comparable between the CON and LC-ISN rats (Table 1). Albumin, ALP, and total cholesterol levels were significantly different between LC and LC-ISN rats, indicating that the values tended to be restored to the levels of CON rats, and liver function might be improved following ISN treatment. The final body weight and relative liver weight (% of body weight) decreased by 50.9% and 31.4%, respectively, and the relative kidney weight (% of body weight) increased by 47.1% in LC rats compared to CON rats. However, these weights, namely the body, liver, and kidney weights were restored to 75.2, 83.4, and 117%, respectively, of CON rats. Microscopy analyses revealed considerable tissue damage in LC rats, including necrosis and fibrosis in the liver and massive cell death in the kidney (Figure 2). In contrast, no tissue abnormalities were found in the liver or kidneys of LC-ISN rats (Figure 2).

### 3.2. Intravenous Administration of Tofacitinib in Rats

**Figure 3:**
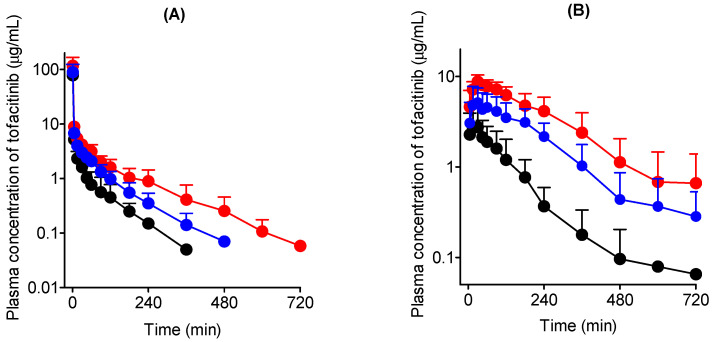
(**A**) Mean arterial plasma concentration–time curves of tofacitinib after 1 min intravenous infusion at a dose of 10 mg/kg to CON (black; *n* = 5), LC (red; *n* = 6) and LC-ISN (blue; *n* = 7) rats. (**B**) Mean arterial plasma concentration–time curves of tofacitinib after oral administration at a dose of 20 mg/kg to CON (*n* = 6), LC (*n* = 7) and LC-ISN (*n* = 7) rats. Bars represent standard deviations. CON, control; LC, liver cirrhosis induced by *N*-dimethylnitrosamine; LC-ISN, LC with treatment of isosakuranetin.

Figure 3A shows the mean arterial plasma concentration–time curves of tofacitinib after intravenous administration at a dose of 10 mg/kg to CON (*n* = 5), LC (*n* = 6), and LC-ISN rats (*n* = 7). The relevant pharmacokinetic parameters of tofacitinib are listed in Table 2. The mean arterial plasma concentration of tofacitinib decreased in a polyexponential manner in all the three rat groups. LC rats showed significantly greater AUC (158%) and longer terminal half-life (219%) for tofacitinib than CON rats. This could be because the CL and CL_NR_ of tofacitnib were significantly slower in LC rats (by 54.4% and 69.9%, respectively) than in CON rats.

In LC-ISN rats, the following changes were observed after tofacitinib administration: (1) Significant increase in AUC (67.5%) and terminal half-life (64.4%) compared to those in CON rats, (2) significant decrease in AUC (35.1%) and terminal half-life (48.5%) compared to those in LC rats, and (3) significantly slower CL and CL_NR_ (42.7% and 54.1%, respectively) than in CON rats, but comparable to those in LC rats.

The percentage of tofacitinib that remained in the gastrointestinal tract at 24 h (GI_24 h_) in LC rats was significantly greater than that in CON and LC-ISN rats. The percentage of tofacitinib excreted in urine as unchanged for 24 h (*Ae*_0–24 h_) was greater by 189% in LC rats and 117% in LC-ISN rats than in CON rats. However, CL_R_ values were comparable among the three groups of rats owing to the significantly greater AUCs in LC and LC-ISN rats. The *V*_ss_ values were also comparable among the three groups.

### 3.3. Oral Administration of Tofacitinib in Rats

Figure 3B shows the mean arterial plasma concentration–time curves of tofacitinib after oral administration at a dose of 20 mg/kg to CON (*n* = 6), LC (*n* = 7), and LC-ISN (*n* = 7) rats, and the relevant pharmacokinetic parameters are listed in Table 3. After oral administration, the plasma concentration of tofacitinib was detected at 5 min (the first blood collection time) for the three groups, indicating that tofacitinib is rapidly absorbed from the gastrointestinal tract.

Pharmacokinetic parameters following oral administration of tofacitinib were as follows: (1) Significant increase in the AUC (429%) and *C*_max_ (158%) in LC rats compared to those in CON rats, (2) increase in *Ae*_0–24 h_ value in LC rats by 203% compared to that in CON rats, (3) significant increase in AUC and *C*_max_ by 223% and 72.2%, respectively, in LC-ISN rats compared to those in CON rats and significant decrease by 38.9% and 33.2%, respectively, compared to those in LC rats, (4) increase in *Ae*_0–24 h_ in LC-ISN rats by 204% compared to CON rats but comparable between LC and LC-ISN rats, and (5) comparable CL_R_ values among the three groups of rats owing to the significantly greater AUCs in LC and LC-ISN rats. *T*_max_ and GI_24 h_ values were also comparable among CON, LC, and LC ISN rats. The *F* values after oral administration were increased by 105% and 92.9% in LC and LC-ISN rats, respectively, compared to that in CON rats.

### 3.4. Tissue Distribution of Tofacitinib

**Figure 4:**
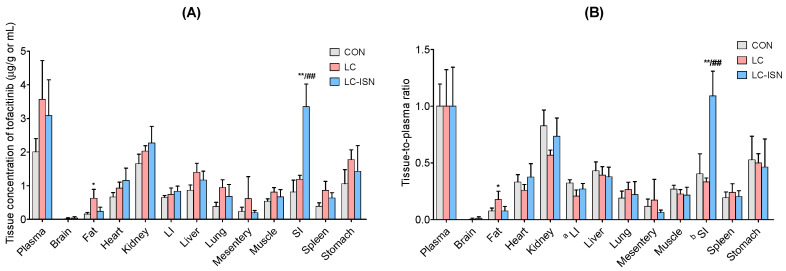
(**A**) Tissue concentration (μg/mL for plasma or μg/g for tissue) and (**B**) tissue-to-plasma (T/P) ratio of tofacitinib at a dose of 10 mg/kg at 30 min after 1 min intravenous infusion to CON, LC and LC-ISN rats (*n* = 3, per group). Bars represent standard deviations. CON, control; LC, liver cirrhosis induced by *N*-dimethylnitrosamine; LC-ISN, LC with treatment of isosakuranetin; LI, large intestine; SI, small intestine. * *p* < 0.05 and ** *p* < 0.01 mean significantly different from CON rats, and ^##^ *p* < 0.01 means significantly different between LC and LC-ISN rats.

Figure 4 shows the tissue concentration (μg/mL for plasma or μg/g for tissue) and tissue-to-plasma (T/P) ratio, 30 min after intravenous administration of 10 mg/kg tofacitinib to CON, LC, and LC-ISN rats (*n* = 3, each). Tofacitinib was widely distributed in all rat tissues in all the three groups. The concentration of tofacitinib in each tissue was comparable among the three groups, except for fat in LC rats and small intestine in LC-ISN rats (Figure 4A). The T/P ratio of tofacitinib was generally low (less than unity) and comparable among the three groups of rats, even though it was significantly higher in the large intestine of LC rats and the small intestine of LC-ISN rats (Figure 4B).

### 3.5. In Vitro Metabolism of Tofacitinib

**Figure 5:**
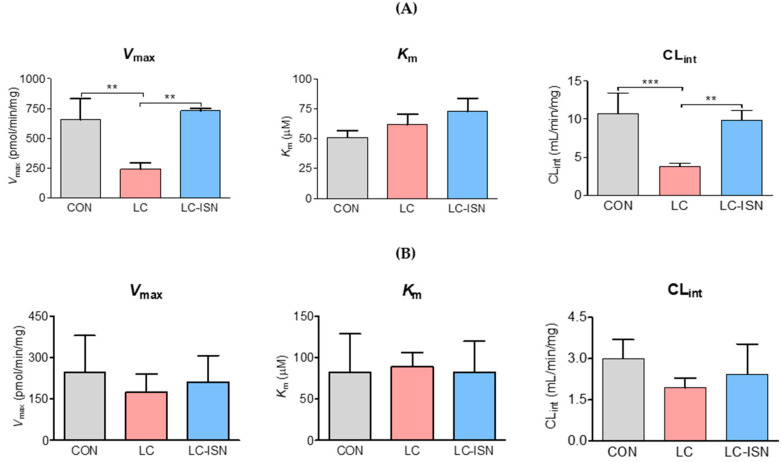
The mean value of *V*_max_, *K*_m_ and CL_int_ for the disappearance of tofacitinib in (**A**) hepatic (*n* = 3, per group) and (**B**) intestinal microsomes (*n* = 3, per group) obtained from CON, LC and LC-ISN rats. This experiment was repeated thrice. Bars represent standard deviations. CON, control; LC, liver cirrhosis induced by *N*-dimethylnitrosamine; LC-ISN, LC with treatment of isosakuranetin; *V*_max_, maximum velocity; *K*_m_, the concentration of tofacitinib at one-half of *V*_max_; CL_int_, intrinsic clearance. ** *p* < 0.01 and *** *p* < 0.001.

Figure 5 shows *V*_max_, *K*_m_, and CL_int_ for the disappearance of tofacitinib in the hepatic (*n* = 3, per group) and intestinal microsomes (*n* = 3, per group) of CON, LC, and LC-ISN rats. In the hepatic microsomes, the *V*_max_ value was significantly slower in LC rats by 63.4% than that in CON rats. However, the value in LC-ISN rats recovered to that in CON rats and consequently significantly increased when compared to that in LC rats. Nonetheless, *K*_m_ values were comparable among the three groups of rats. As a result, hepatic CL_int_ value decreased by 64.9% in LC rats compared to that in CON rats; the value in LC-ISN rats recovered to 92.2% of CON rats (Figure 5A). In contrast, CL_int_ in the intestine of LC rats tended to be slower than those in CON and LC-ISN rats, but was not significantly different among the three groups of rats owing to the great interspecies differences. *V*_max_ and *K*_m_ values in the intestinal microsomes were also comparable among the three groups of rats (Figure 5B).

### 3.6. Expression of CYP Isoforms and Other Involved Proteins in the Hepatic and Intestinal Microsomes

**Figure 6:**
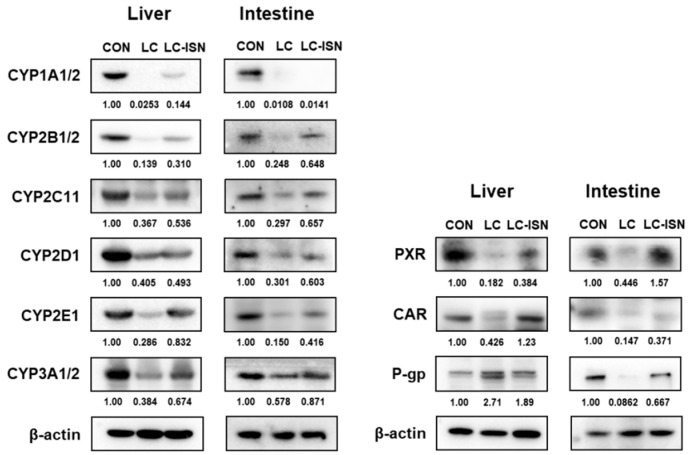
Immunoblot analyses of CYP isozymes, PXR, CAR and P-gp in the hepatic and intestinal microsomes obtained from CON, LC and LC-ISN rats. ß-actin was used for the loading control. This experiment was repeated thrice. Band density was determined by ImageJ 1.45s software (NIH). CON, control; LC, liver cirrhosis induced by *N*-dimethylnitrosamine; LC-ISN, LC with treatment of isosakuranetin; CYP, cytochrome P450; PXR, pregnane X receptor; CAR, constitutive androstane receptor; P-gp, P-glycoprotein.

Figure 6 shows the hepatic and intestinal expression of CYP isoforms, PXR, CAR, and P-gp in CON, LC, and LC-ISN rats. The protein expression of CYP2C11 in the hepatic and intestinal microsomes of LC rats decreased by 63.3% and 70.3%, respectively, compared to those in CON rats, but increased by 46.0% and 121%, respectively, in LC-ISN rats compared to those in LC rats. The expression of hepatic and intestinal CYP3A1/2 was also reduced by 61.6% and 42.2%, respectively, in LC rats compared to those in CON rats, but recovered to 67.4% and 87.1% of CON rats, respectively, in LC-ISN rats. Generally, the protein expression of CYP1A1/2, CYP2B1/2, CYP2D1 and CYP2E1 in the hepatic and intestinal microsomes decreased in LC rats compared to those in CON rats, but increased in LC-ISN rats compared to those in LC rats. Additionally, the expression of PXR and CAR, nuclear xenobiotic receptors, was significantly decreased in the hepatic and intestinal microsomes of LC rats compared to those in CON rats. However, following ISN treatment, the expression of PXR and CAR in LC-ISN rats recovered to that in CON rats. Nevertheless, the expression of hepatic P-gp increased by 171% in LC rats compared to that in CON rats and then decreased by 30.3% in LC-ISN rats compared to that in LC rats. The expression of intestinal P-gp decreased by 91.4% in LC rats compared to that in CON rats and recovered to 66.7% of that in CON rats following ISN treatment.

## 4. Discussion

In a preliminary study, DMN induced LC, by increasing GOT, GPT, total cholesterol, and ALP levels and by reducing final body weight, total protein, albumin, and liver weight; however, ISN protected the liver from cirrhosis and improved liver function compared to LC rats. The parameters for liver function in LC-ISN rats recovered to levels similar to those in CON rats. Furthermore, decreased kidney function, such as CL_CR_ and relative kidney weight (% of body weight) in LC rats, improved in LC-ISN rats following ISN administration; little tissue damage such as fibrosis, necrosis, and massive cell death induced by DMN were observed in LC-ISN rats, suggesting that ISN could have a protective effect on both the liver and kidney during the development of LC by DMN. Similar results have been reported for flavanones such as poncirin [24] and naringenin [22] having hepatoprotective effects.

The AUC ratios based on tofacitinib dose were not proportional to the intravenous dose over 20 mg/kg and the oral dose over 50 mg/kg [13]. Therefore, 10 and 20 mg/kg tofacitinib were chosen for intravenous and oral administration, respectively. After intravenous administration of 10 mg/kg tofacitinib to LC rats, the AUC of tofacitinib was higher than that of CON rats owing to slower CL. The significantly slower CL in LC rats was attributed to a slower CL_NR_. The percentage of gastrointestinal excretion including biliary excretion was very low; the GI_24 h_ values ranged from 0.164% to 2.07% of the intravenous dose for all three groups of rats. The low GI_24 h_ values was not due to the chemical or enzymatic degradation of tofacitinib in the gastrointestinal tract of rats, as tofacitinib was stable at various pH buffers (pH 2–10) and gastric juice (pH 3.5) for 24 h [28]. Moreover, the biliary excretion of tofacitinib after intravenous administration at a dose of 10 mg/kg in rats (*n* = 3) was 0.703% of the unchanged drug dose [13]. Therefore, the contribution of gastrointestinal excretion to CL_NR_ was almost negligible, and CL_NR_ in Table 2 represents the metabolic clearance of tofacitinib. Therefore, *Ae*_0-24 h_ was greater because of the slower CL_NR_ in LC rats. However, the CL_R_ was comparable between CON and LC rats because of the significantly greater AUC in LC rats. After intravenous administration of tofacitinib to LC-ISN rats, the AUC of tofacitinib was significantly lower and the terminal half-life was shorter than those in LC rats owing to the faster CL in LC-ISN rats than in LC rats. This indicates that ISN protects the liver from DMN-induced cirrhosis and improves hepatic metabolic function.

The slower CL_NR_ in LC rats was supported by a significant decrease in the in vitro hepatic CL_int_ of tofacitinib and slower hepatic blood flow rate [36] in LC rats compared to CON rats. The free fraction of tofacitinib did not affect CL_NR_ because the plasma protein binding of tofacitinib was generally low (18.0–25.1%), even though plasma protein binding was significantly decreased in LC rats. Tofacitinib has an intermediate hepatic extraction ratio (30–70%) because the hepatic first-pass metabolism after absorption into the portal vein was 42.0% in rats [13]. Furthermore, hepatic CL_int_ of tofacitinib in LC-ISN rats was significantly faster following ISN treatment than that in LC rats. Therefore, the slower hepatic CL_int_ in LC rats was mainly due to a decrease in CYP3A1/2 and CYP2C11 proteins, the main metabolic enzymes for tofacitinib, in LC rats, whereas the faster hepatic CL_int_ in LC-ISN rats was due to an increase in CYP3A1/2 and CYP2C11. The protein expression of hepatic CYP3A and CYP2C were also decreased in LC rats induced by CCl_4_ [37]. Decreased protein expression of CYP3A1/2 and CYP2C11 in LC rats was also recovered by ISN treatment through the upregulation of CAR and PXR transcription factors [38]. It has been reported that the mRNA expression of PXR was reduced in LC mice and rats induced by CCl_4_ [39], while the mRNA expression of CAR decreased in patients with stage 3 liver fibrosis [40]. Based on these results, reduced PXR and CAR expression may affect the transcription of CYP isoforms, resulting in decreased activity or expression of hepatic CYP2C11 and CYP3A1/2.

Notably, the protein expression of hepatic P-gp was significantly higher in LC rats than in CON rats. Tofacitinib is a substrate of P-gp [41]. Overexpression of efflux transporter proteins in the liver generally increases *V*_ss_ of drugs [42]. Although *V*_ss_ of tofacitinib in LC rats was not significantly different from that in CON rats, the *V*_ss_ of tofacitinib increased by 86.0% in LC rats probably due to the higher protein expression of hepatic P-gp in LC rats. The *V*_ss_ value of digoxin increased in rats with non-alcoholic fatty liver disease owing to enhanced hepatic P-gp expression, leading to changes in digoxin pharmacokinetics [43]. Hepatic P-gp expression slightly decreased in LC-ISN rats compared to LC rats but was increased compared to CON rats, which could be attributed to the increased *V*_ss_ in LC-ISN rats compared to CON rats.

After oral administration of 20 mg/kg tofacitinib to LC rats, the AUC of tofacitinib was greater than that of CON rats. The absorption of tofacitinib from the gastrointestinal tract was almost complete; GI_24 h_ values were less than 1.73% of the oral dose in the three groups of rats. This indicates that the complete absorption of tofacitinib for 24 h after oral administration was not the main reason for the increased AUCs in LC. Several reasons for the greater AUC in LC rats have been considered. First, the protein expression of P-gp decreased in the intestine of LC rats, indicating that the reduced expression of P-gp increased tofacitinib absorption in LC rats. Similarly, the AUC of zidovudine for anti-HIV therapy increased compared to control rats owing to the decreased expression and function of intestinal P-gp in rats with acute liver failure induced by thioacetamide [44]. Second, the considerably slower intestinal CL_int_ and decreased intestinal expression of CYP3A1/2 and CYP2C11 are other reasons for the greater AUC in LC rats. Lastly, even though this has not been validated in rats, alterations in intestinal tight junction proteins may increase the intestinal permeability of drugs [45] and thus might increase the AUC of tofacitinib. This was supported by the fact that the intestinal permeability of Rhodamine 123 increased in acute liver failure rats; thus, the cumulative absorption of Rhodamine 123 increased compared to control rats [44].

However, the AUC of tofacitinib after oral administration in LC rats increased by 429% compared to CON rats, which was greater than 158% increase after intravenous administration. These results could be due to the considerably decreased intestinal CL_int_ via decreased expression of CYP3A1/2 and CYP2C11 in the intestine of LC rats and slower metabolism in the liver of LC rats. Furthermore, the expression of intestinal P-gp was almost completely inhibited in LC rats, which appeared to affect the increased absorption of the drug. Similar results were also reported that the increase in AUC of telithromycin, a ketolide antibiotic, after oral administration to inflammatory rats induced by lipopolysaccharide was considerably greater than AUC increase after intravenous administration, which could have been due to a decrease in intestinal metabolism of telithromycin by decreased CYP3A1/2 expression as well as a decreased hepatic metabolism of telithromycin in inflammatory rats [46].

After oral administration of tofacitinib to LC-ISN rats, the AUC of tofacitinib significantly decreased by 38.9% compared to that in LC rats. This could be due to the recovery of enzyme activity in the intestine and liver. Decreased expression of intestinal CYP3A1/2 and CYP2C11 proteins in LC rats was also restored by ISN treatment through the upregulation of PXR and/or CAR transcription factors [38,47,48]. Based on these results, increased PXR and/or CAR may affect the expression of CYP isoforms, resulting in increased activity or expression of intestinal CYP2C11 and CYP3A1/2 in LC-ISN rats. Furthermore, increased P-gp expression in the intestine of LC-ISN rats appeared to play a role in the reduced plasma concentration of tofacitinib compared to LC rats. However, the hepatoprotective mechanisms of ISN remain unknown. It has been reported that poncirin, isosakuranetin-7-O-neohesperidoside, protects the liver in LC mice induced by CCl_4_ through attenuating inflammatory cytokines and oxidative stress [24]. Naringenin, a flavanone derivative, also has anti-inflammatory and antioxidative effects [49]. ISN, a flavanone derivative and aglycon of poncirin, possess antioxidant and anti-inflammatory properties [19]. Therefore, it is presumed that ISN has hepatoprotective mechanisms similar to those of poncirin and naringenin and restored the CYP isoforms through the up-reulgation of PXR and CAR in LC rats. However, due to the limitation of study design, it was not confirmed whether an increase in the expression of CYP isozyme, PXR and CAR occurred in CON rats treated with ISN for 6 weeks. Further studies are needed to access the effects of ISN in CON rats.

## 5. Conclusions

The AUC of tofacitinib was significantly higher in LC rats than that in CON rats and significantly lower in LC-ISN rats than that in LC rats owing to a decrease in CL_NR_ in LC rats and increased hepatic CYP3A1/2 and CYP2C11 activity in LC-ISN rats, respectively. After oral administration of tofacitinib, the pattern of AUC changes in LC and LC-ISN rats was similar to that observed after intravenous administration. This could also be due to the decreased intestinal CYP3A1/2, CYP2C11 and P-gp expression in LC rats and increased intestinal CYP3A1/2, CYP2C11 and P-gp protein levels in LC-ISN rats, respectively, as well as in the liver. The decreased expression of hepatic and intestinal CYP3A1/2 and CYP2C11 proteins in LC was recovered by ISN treatment through the upregulation of PXR and/or CAR in LC-ISN rats.

## Figures and Tables

**Table 1:** The mean values (±standard deviation) of initial and final body weights, plasma concentrations of total protein, albumin, urea nitrogen, GOT, GPT, ALP, total cholesterol, S_CR_, CL_CR_, and relative liver and kidney weights in CON, LC and LC-ISN rats.

Parameters	CON (*n* = 3)	LC (*n* = 3)	LC-ISN (*n* = 3)
Body weight (g)			
Initial	109 ± 1.15	105 ± 3.06	105 ± 2.65
Final	383 ± 14.9	188 ± 1.15 **	288 ± 67.8
Plasma			
Total protein (g/dL)	6.85 ± 0.288	4.09 ± 0.731 **	6.17 ± 0.466 ^##^
Albumin (g/dL)	3.74 ± 0.407	2.09 ± 0.484 **	3.24 ± 0.205 ^#^
Urea nitrogen (mg/dL)	11.7 ± 1.14	19.1 ± 6.58	13.0 ± 2.00
GOT (IU/L)	39.3 ± 12.3	111 ± 19.0 **	69.5 ± 19.5
GPT (IU/L)	20.0 ± 2.03	47.3 ± 11.3 *	36.0 ± 6.22
ALP (K-AU)	50.8 ± 6.68	282 ± 89.6 **	118 ± 17.5 ^#^
Total cholesterol (mg/dL)	77.2 ± 6.22	142 ± 22.6 **	81.1 ± 11.5 ^##^
S_CR_ (mg/dL)	0.570 ± 0.0942	0.597 ± 0.0869	0.667 ± 0.0415
CL_CR_ (mL/min/kg)	3.65 ± 0.823	1.71 ± 0.778 *	2.43 ± 0.391
Liver weight (% of body weight)	3.25 ± 0.263	2.23 ± 0.490 *	2.71 ± 0.257
Kidney weight (% of body weight)	0.734 ± 0.0279	1.08 ± 0.0379 **	0.856 ± 0.124 ^#^
Plasma protein binding (%)	25.1 ± 2.75	18.0 ± 1.18 **	19.7 ± 1.21 *

GOT, glutamate oxaloacetate transaminase; GPT, glutamate pyruvate transaminase; ALP, alkaline phosphatase; S_CR_, serum creatinine concentration; CL_CR_, creatinine clearance; CON, control; LC, liver cirrhosis induced by *N*-dimethylnitrosamine; LC-ISN, LC with treatment of isosakuranetin. * *p* < 0.05 and ** *p* < 0.01 mean significantly different from CON rats, and ^#^ *p* < 0.05 and ^##^ *p* < 0.01 mean significantly different between LC and LC-ISN rats.

**Table 2:** Pharmacokinetic parameters of tofacitinib after 1 min intravenous infusion at a dose of 10 mg/kg toracitinib to CON, LC and LC-ISN rats. Data are shown as mean ± standard deviation.

Parameters	CON (*n* = 5)	LC (*n* = 6)	LC-ISN (*n* = 7)
Body weight (g)	430 ± 20.0	249 ± 67.6 ***	309 ± 30.6 ***
Terminal half-life (min)	35.4 ± 19.2	113 ± 43.7 **	58.2 ± 16.8 ^#^
AUC (μg∙min/mL)	440 ± 211	1135 ± 391 **	737 ± 107 ^#^
CL (mL/min/kg)	24.1 ± 6.27	11.0 ± 2.06 ***	13.8 ± 1.73 ***
CL_R_ (mL/min/kg)	3.73 ± 1.09	4.65 ± 2.74	4.10 ± 1.64
CL_NR_ (mL/min/kg)	21.1 ± 4.57	6.35 ± 1.27 ***	9.68 ± 2.40 ***
*V*_ss_ (mL/kg)	429 ± 253	798 ± 177	800 ± 326
*Ae*_0–24 h_ (% of dose)	13.9 ± 2.28	40.2 ± 18.9 *	30.1 ± 12.3
GI_24 h_ (% of dose)	0.164 ± 0.102	2.07 ± 2.12	0.166 ± 0.135 ^#^

CON, control; LC, liver cirrhosis induced by *N*-dimethylnitrosamine; LC-ISN, LC with treatment of isosakuranetin. * *p* < 0.05, ** *p* < 0.01 and *** *p* < 0.001 mean significantly different from CON rats, and ^#^ *p* < 0.05 means significantly different between LC and LC-ISN rats.

**Table 3:** Pharmacokinetic parameters of tofacitinib after oral administration at a dose of 20 mg/kg tofacitinib to CON, LC and LC-ISN rats. Data are shown as mean ± standard deviation.

Parameters	CON (*n* = 6)	LC (*n* = 7)	LC-ISN (*n* = 7)
Body weight (g)	386 ± 28.2	222 ± 36.6 ***	255 ± 38.6 ***
AUC (μg∙min/mL)	384 ± 122	2031 ± 500 ***	1240 ± 128 ***^,###^
*C*_max_ (μg/mL)	3.53 ± 1.46	9.10 ± 1.26 ***	6.08 ± 1.99 *^,##^
*T*_max_ (min)	50.0 ± 44.2	55.7 ± 36.4	85.7 ± 79.7
CL_R_ (mL/min/kg)	6.98 ± 2.99	3.62 ± 1.78	5.71 ± 2.47
*Ae*_0–24 h_ (% of dose)	11.8 ± 4.08	35.8 ± 16.9 *	35.9 ± 16.8 *
GI_24 h_ (% of dose)	0.106 ± 0.101	1.73 ± 2.04	0.340 ± 0.288
*F* (%)	43.6	89.5	84.1

CON, control; LC, liver cirrhosis induced by *N*-dimethylnitrosamine; LC-ISN, LC with treatment of isosakuranetin. * *p* < 0.05 and *** *p* < 0.001 mean significantly different from CON rats, and ^##^ *p* < 0.01 and ^###^ *p* < 0.001 mean significantly different between LC and LC-ISN rats.

## Data Availability

Not applicable.

## References

[1] Claxton L., Taylor M., Soonasra A., Bourret J.A., Gerber R.A. (2018). An economic evaluation of tofacitinib treatment in rheumatoid arthritis after methotrexate or after 1 or 2 TNF inhibitors from a U.S. payer perspective. J. Manag. Care Spec. Pharm..

[2] Palmroth M., Kuuliala K., Peltomaa R., Virtanen A., Kuuliala A., Kurttila A., Kinnunen A., Leirisalo-Repo M., Silvennoinen O., Isomäki P. (2021). Tofacitinib Suppresses Several JAK-STAT Pathways in Rheumatoid Arthritis In Vivo and Baseline Signaling Profile Associates With Treatment Response. Front. Immunol..

[3] Hu X., Li J., Fu M., Zhao X., Wang W. (2021). The JAK/STAT signaling pathway: From bench to clinic. Signal Transduct. Target. Ther..

[4] Sandborn W.J., Peyrin-Biroulet L., Sharara A.I., Su C., Modesto I., Mundayat R., Gunay L.M., Salese L., Sands B.E. (2022). Efficacy and safety of tofacitinib in ulcerative colitis based on prior tumor necrosis factor inhibitor failure status. Clin. Gastroenterol. Hepatol..

[5] Fukuda T., Naganuma M., Kanai T. (2019). Current new challenges in the management of ulcerative colitis. Intest. Res..

[6] Bachelez H., Van de Kerkhof P.C., Strohal R., Kubanov A., Valenzuela F., Lee J.H., Gupta P. (2015). Tofacitinib versus etanercept or placebo in moderate-to-severe chronic plaque psoriasis: A phase 3 randomised non-inferiority trial. Lancet.

[7] Papp K.A., Menter M.A., Abe M., Elewski B., Feldman S.R., Gottlieb A.B., Langley R., Luger T., Thaci D., Buonanno M. (2015). OPT Pivotal 1 and OPT Pivotal 2 investigators. Tofacitinib, an oral Janus kinase inhibitor, for the treatment of chronic plaque psoriasis: Results from two randomized, placebo-controlled, phase III trials. Br. J. Dermatol..

[8] Hogan S., Wang S., Ibrahim O., Piliang M., Bergfeld W. (2019). Long-term treatment with tofacitinib in severe alopecia areata: An update. J. Clin. Aesthet. Dermatol..

[9] Levy L.L., Urban J., King B.A. (2015). Treatment of recalcitrant atopic dematitis with the oral Janus kinase inhibitor tofacitinib citrate. J. Am. Acad. Dermatol..

[10] Mohanakrishnan R., Beier S., Deodhar A. (2022). Tofacitinib for the treatment of active ankylosing spondylitis in adults. Expert Rev. Clin. Immunol..

[11] Dowty M.E., Lin J., Ryder T.F., Wang W., Walker G.S., Vaz A., Prakash C. (2014). The pharmacokinetics, metabolism, and clearance mechanisms of tofacitinib, a janus kinase inhibitor, in humans. Drug Metab. Dispos..

[12] Bannwarth B., Kostine M., Poursac N. (2013). A pharmacokinetic and clinical assessment of tofacitinib for the treatment of rheumatoid arthritis. Expert Opin. Drug Metab. Toxicol..

[13] Lee J.S., Kim S.H. (2019). Dose-dependent pharmacokinetics of tofacitinib in rats: Influence of hepatic and intestinal first-pass metabolism. Pharmaceutics.

[14] Radovanović-Dinić B., Tešić-Rajković S., Zivkovic V., Grgov S. (2018). Clinical connection between rheumatoid arthritis and liver damage. Rheumatol. Int..

[15] Yang X., Meng Y., Han Z., Ye F., Wei L., Zong C. (2020). Mesenchymal stem cell therapy for liver disease: Full of chances and challenges. Cell biosci..

[16] Pinter M., Trauner M., Peck-Radosavljevic M., Sieghart W. (2016). Cancer and liver cirrhosis: Implications on prognosis and management. ESMO Open..

[17] Peterson J.J., Dwyer J.T., Beecher G.R., Bhagwat S.A., Gebhardt S.E., Haytowitz D.B., Holden J.M. (2006). Flavanones in oranges, tangerines (mandarins), tangors, and tangelos: A compilation and review of the data from the analytical literature. J. Food Compos. Anal..

[18] Straub I., Krügel U., Mohr F., Teichert J., Rizun O., Konrad M., Oberwinkler J., Schaefer M. (2013). Flavanones that selectively inhibit TRPM3 attenuate thermal nociception in vivo. Mol. Pharmacol..

[19] Jia S., Zhang Y., Yu J. (2017). Antinociceptive effects of isosakuranetin in a rat model of peripheral neuropathy. Pharmacology.

[20] Lee U., Oh E. (2015). Pharmacokinetic changes of drugs in a rat model of liver cirrhosis induced by dimethylnitrosamine, alone and in combination with diabetes mellitus induced by streptozotocin. Biopharm. Drug Dispos..

[21] Bae S.K., Lee S.J., Kim T., Kim J.W., Lee I., Kim S.G., Lee M.G. (2006). Pharmacokinetics and therapeutic effects of oltipraz after consecutive or intermittent oral administration in rats with liver cirrhosis induced by dimethylnitrosamine. J. Pharm. Sci..

[22] Lee M.H., Yoon S., Moon J.O. (2004). The flavonoid naringenin inhibits dimethylnitrosamine-induced liver damage in rats. Biol. Pharm. Bull..

[23] Chooi K.F., Kuppan Rajendran D.B., Phang S.S., Toh H.H. (2016). The dimethylnitrosamine induced liver fibrosis model in the rat. J.Vis. Exp..

[24] Ullah H., Khan A., Baig M.W., Ullah N., Ahmed N., Tipu M.K., Ali H., Khan S. (2020). Poncirin attenuates CCl4-induced liver injury through inhibition of oxidative stress and inflammatory cytokines in mice. BMC complement. Med. Ther..

[25] Kim S.H., Lee J.S., Lee M.G. (1999). Stability, blood partition and plasma protein binding of ipriflavone, an isoflavone derivative. Biopharm. Drug Dispos..

[26] Svein Ø., Theodor W.G. (1982). Comparison of equilibrium time in dialysis experiments using spiked plasma or spiked buffer. J. Pharm. Sci..

[27] Shin W.G., Lee M.G., Lee M.H., Kim N.D. (1991). Factors influencing the protein binding of vancomycin. Biopharm. Drug Dispos..

[28] Kim J.E., Park M.Y., Kim S.H. (2020). Simple determination and quantification of tofacitinib, a JAK inhibitor, in rat plasma, urine and tissue homogenates by HPLC and its application to a pharmacokinetic study. J. Pharm. Investig..

[29] Park H.J., Bae S.H., Kim S.H. (2021). Dose-independent pharmacokinetics of loganin in rats: Effect of intestinal first-pass metabolism on bioavailability. J. Pharm. Investig..

[30] Gwak E.H., Yoo H.Y., Kim S.H. (2020). Effects of diabetes mellitus on the disposition of tofacitinib, a Janus kinase inhibitor, in rats. Biomol. Ther..

[31] Bae S.H., Chang S.Y., Kim S.H. (2020). Slower elimination of tofacitinib in acute renal failure rat Models: Contribution of hepatic metabolism and renal excretion. Pharmaceutics.

[32] Duggleby R.G. (1995). Analysis of enzyme progress curves by nonlinear regression. Methods Enzymol..

[33] Bae S.H., Park J.H., Choi H.G., Kim H., Kim S.H. (2018). Imidazole antifungal drugs inhibit the cell proliferation and invasion of human breast cancer cells. Biomol. Ther (Seoul)..

[34] Gibaldi M., Perrier D. (1982). Pharmacokinetics.

[35] Chiou W.L. (1978). Critical evaluation of the potential error in pharmacokinetic studies of using the linear trapezoidal rule method for the calculation of the area under the plasma level-time curve. J. Pharmacokinet. Biopharm..

[36] Goeting N.L., Fleming J.S., Gallagher P., Walmsely B.H., Karran S.J. (1986). Alterations in liver blood flow and reticuloendothelial function in progressive cirrhosis in the rat. J. Nucl. Med..

[37] Bastien M.C., Leblond F., Pichette V., Villeneuve J.P. (2000). Differential alteration of cytochrome P450 isoenzymes in two experimental models of cirrhosis. Can. J. Physiol. Pharmacol..

[38] Burk O., Koch I., Raucy J., Hustert E., Eichelbaum M., Brockmöller J., Zanger U.M., Wojnowski L. (2004). The induction of cytochrome P450 3A5 (CYP3A5) in the human liver and intestine is mediated by the xenobiotic sensors pregnane X receptor (PXR) and constitutively activated receptor (CAR). J. Biol. Chem..

[39] Hanada K., Nakai K., Tanaka H., Suzuki F., Kumada H., Ohno Y., Ozawa S., Ogata H. (2012). Effect of nuclear receptor downregulation on hepatic expression of cytochrome P450 and transporters in chronic hepatitis C in association with fibrosis development. Drug Metab. Pharmacokinet..

[40] Daujat-Chavanieu M., Gerbal-Chaloin S. (2020). Regulation of CAR and PXR expression in health and disease. Cells.

[41] Hussa D.A. (2014). 2013 new drug update: What do new approvals hold for the elderly?. Consult. Pharm..

[42] Grover A., Benet L.Z. (2009). Effects of drug transporters on volume of distribution. AAPS J..

[43] Jeong H.J., Lee S.H., Kang H.E. (2021). Changes in digoxin pharmacokinetics associated with hepatic P-glycoprotein upregulation in rats with non-alcoholic fatty liver disease. Fundam. Clin. Pharmacol..

[44] Wang F., Miao M.X., Sun B.B., Wang Z.J., Tang X.G., Chen Y., Zhao K.J., Liu X.D., Liu L. (2017). Acute liver failure enhances oral plasma exposure of zidovudine in rats by downregulation of hepatic UGT2B7 and intestinal P-gp. Acta. Pharmacol. Sin..

[45] Aguirre Valadez J.M., Rivera-Espinosa L., Méndez-Guerrero O., Chávez-Pacheco J.L., García Juárez I., Torre A. (2016). Intestinal permeability in a patient with liver cirrhosis. Ther. Clin. Risk Manag..

[46] Lee J.H., Cho Y.K., Jung Y.S., Kim Y.C., Lee M.G. (2008). Effects of Escherichia coli lipopolysaccharide on telithromycin pharmacokinetics in rats: Inhibition of metabolism via CYP3A. Antimicrob. Agents. Chemother..

[47] He L., Zhou X., Huang N., Li H., Li T., Yao K., Tian Y., Hu C.A., Yin Y. (2017). Functions of pregnane X receptor in self-detoxification. Amino acids..

[48] Kobayashi K., Kuze J., Abe S., Takehara S., Minegishi G., Igarashi K., Kitajima S., Kanno J., Yamamoto T., Oshimura M. (2019). CYP3A4 induction in the liver and intestine of pregnane X receptor/CYP3A-Humanized Mice: Approaches by mass spectrometry imaging and portal blood analysis. Mol. Pharmacol..

[49] Hernández-Aquino E., Muriel P. (2018). Beneficial effects of naringenin in liver diseases: Molecular mechanisms. World J. Gastroenterol..

